# Heat shock transcription factor 2 reduces mitochondrial pathway apoptosis in intestinal epithelial cells by inhibiting the increase in mitochondrial membrane permeability in ulcerative colitis

**DOI:** 10.1371/journal.pone.0325275

**Published:** 2025-05-29

**Authors:** Wen Wang, Yunling Wen, Juan Luo, Yinglei Miao, Fengrui Zhang, Junkun Niu

**Affiliations:** Department of Gastroenterology, First Affiliated Hospital of Kunming Medical University, Yunnan Institute of Digestive Diseases, Kunming, Yunnan, China; Sungkyunkwan University - Suwon Campus: Sungkyunkwan University - Natural Sciences Campus, KOREA, REPUBLIC OF

## Abstract

The destruction of intestinal mucosal mechanical barrier homeostasis caused by excessive apoptosis of intestinal epithelial cells (IECs) is an important reason for the occurrence and development of ulcerative colitis (UC). The increase in mitochondrial membrane permeability caused by the opening of the mitochondrial membrane permeability transition pore (mPTP) is a key link in the initiation of mitochondrial pathway apoptosis. Our previous studies revealed that heat shock transcription factor 2 (HSF2), which is highly expressed in the intestinal mucosa of UC patients, can inhibit the expression of the cytochrome C (Cyto-C)/Caspase-9/Caspase-3 proteins in the mitochondrial pathway of apoptosis, but the regulatory mechanism is unknown. It has been reported that heat shock proteins regulated by heat shock transcription factors are closely related to mPTP opening. Therefore, we hypothesized that HSF2 affects mitochondrial pathway apoptosis in IECs by regulating mPTP opening. In this study, we altered the level of HSF2 in Caco-2 cells by lentivirus transfection to explore the changes in the mitochondrial membrane permeability of Caco-2 cells in an inflammatory environment. Subsequently, the mPTP agonist atractylorhizin (Atr) and inhibitor cyclosporine A (CsA) were used to clarify the regulatory effects of HSF2 on mPTP and the Cyto-C/Caspase-9/Caspase-3 pathways. Our study confirmed for the first time that HSF2 plays a protective role in UC by inhibiting mPTP opening, the increase in mitochondrial membrane permeability and the activation of the mitochondrial-mediated apoptosis pathway in IECs.

## Introduction

Ulcerative colitis (UC) is a chronic, nonspecific, inflammatory disease mainly involving the intestinal tract [1]. Its incidence and prevalence are increasing annually worldwide [2], but its pathogenesis is not completely clear. There is a lack of effective drugs for treating UC, and the existing treatment methods are often accompanied by side effects. The clinical efficacy of UC treatment is often unsatisfactory, and the cost is high, which places great economic pressure and psychological burdens on patients and their families [3]. This has become an urgent medical and social problem to be solved.

Although the mechanism of UC is not completely clear and based on a multifactorial pathogenesis, it is currently generally believed to be closely related to damage to the intestinal mucosal mechanical barrier [1,4]. The mechanical barrier of the intestinal mucosa is a physical protective layer composed of intestinal epithelial cells (IECs) and intercellular connections. IECs are the basic structural unit of the intestinal mucosa mechanical barrier and are among the cells with the highest renewal rates [5]. The intestinal mucosal mechanical barrier is usually in a steady state of alternating growth and death of IECs, and the occurrence and development of UC is often accompanied by the destruction of this steady state [6].

The inflammatory state of the intestine and destruction of intestinal mucosal mechanical barrier homeostasis in UC cause a state of stress in the body, resulting in the production of many stress proteins to fight the damage. Heat shock proteins (HSPs) are among these proteins [7]. Heat shock transcription factors (HSFs) regulate the transcription of HSPs. Our previous study revealed that the HSF2 protein is upregulated in the intestinal mucosa of active UC patients compared with that in healthy controls [8,9]. It has been confirmed that HSF2 can inhibit activation of the NLRP3 inflammasome, reduce the secretion of IL-1β, and play a protective role in UC through anti-inflammatory effects [10]. What’s more, HSF2 can inhibit mitochondrial pathway apoptosis and maintain the homeostasis of the intestinal mucosal mechanical barrier [11]. Moreover, our previous study revealed that overexpression of HSF2 can reduce the number of damaged mitochondria in a Caco-2 cell inflammation model [12].

Mitochondria are energy factories and control various types of programmed cell death [13], including mitochondrial pathway apoptosis [14]. During this process of apoptosis, changes in mitochondrial membrane permeability are key [15]. Mitochondrial membrane permeability is mainly determined by the opening and closing of the mitochondria permeability transition pore (mPTP). The mPTP is also an ion channel that controls material and charge exchanges between the inside and outside of mitochondria [16]. When cells are stimulated by endogenous apoptotic signals, the proapoptotic molecule cytochrome C (Cyto-C) is released from the inner mitochondrial membrane into the cytoplasm via the mPTP, which is an initial important step in mitochondrial pathway apoptosis [17]. Cyto-C can form an apoptotic complex with Apaf-1 and Caspase-9 in the cytoplasm, which in turn activates Caspase-3 and initiates downstream cascades. Moreover, Cyto-C is also a very important electron transporter, and its release can cause a change in the electrochemical potential on both sides of the mitochondrial inner membrane, which manifests as a decrease in the mitochondrial membrane potential (MMP) in the early stage of apoptosis [18]. Therefore, the state and degree of opening and closing of the mPTP directly affect the release of Cyto-C, the change in the MMP and the activation of the mitochondrial pathway of apoptosis. The degree of mPTP opening is affected by many factors, including its structure and HSPs [19]. Moreover, HSFs are important transcription factors that regulate HSP expression. HSF2 can bind to the chromatin structure of the HSP27 and HSP90 promoters and regulate their expression [20]. In addition, our previous study revealed that HSF2 plays a protective role in UC by inhibiting the mitochondrial pathway to reduce excessive apoptosis of IECs, but the mechanism is not clear [11]. Therefore, we hypothesized that HSF2 may inhibit mitochondrial pathway apoptosis in IECs by reducing the degree of mPTP opening and promoting the stability of mitochondrial membrane permeability. In this study, experiments at the cellular level were carried out, which confirmed our theory.

## Materials and methods

### 1. Cell culture

The Caco-2 cells used in this study were purchased from the Cell Bank of Kunming Institute of Zoology, Chinese Academy of Sciences. The cells were cultured in Dulbecco’s modified Eagle’s medium (DMEM, HyClone, NY, USA) supplemented with 10% fetal bovine serum (HyClone) in 5% CO_2_ at 37 °C. HSF2 interference (HSF2 RNAi lentivirus) and overexpression (LV-HSF2 lentivirus) lentiviruses were used to reduce and increase the levels of HSF2 gene transcription and protein expression, respectively, in Caco-2 cells. The lentiviruses were purchased from Shanghai GeneChem Co., China, and the transfection method was the same as that used in our previous study [11].

### 2. Construction of the disease model

The Caco-2 cell model of inflammation was induced by treatment with 200 ng/ml LPS for 24 h.

### 3. Assessment of mPTP opening

The Caco-2 cells were digested with trypsin-EDTA solution to prepare a cell suspension at a final concentration of 1x10^6^ cells/ml, and subsequently, 1 ml of the cell suspension was added to the flow cytometry tube. In accordance with the instructions of the mPTP assay kit (Abcam, UK), 5 µl of mPTP staining solution and 5 µl of CoCl_2_ solution were added to the flow cytometry tube, followed by thorough mixing. Both mPTP staining solution and CoCl_2_ solution were supplied by the mPTP assay kit. The cells were incubated at 37 °C in the dark for 15 min. After incubation, the cells were collected by centrifugation at 1200 rpm for 6 min at room temperature. One milliliter of detection buffer was added to each sample, followed by gentle resuspending. The samples were placed on ice and then immediately (< 1 h) examined and analyzed by flow cytometry.

Atractylorhizin (Atr, 20μM, 24h) and cyclosporine A (CsA, 0.5μM, 24h) are agonists and inhibitors of the mPTP, respectively.

### 4. Assessment of the MMP

JC-1 was a fluorescent probe widely used for detecting changes in MMP. When the MMP was high, JC-1 accumulated in the mitochondrial matrix to form polymers, which could produce red fluorescence. When the MMP was low, JC-1 could not accumulate in the mitochondrial matrix. At this time, JC-1 was a monomer and could produce green fluorescence. In this way, it was convenient to detect the changes in MMP through the transformation of fluorescence color. The maximum emission wavelength of JC-1 monomer was 530nm and the maximum emission wavelength of JC-1 polymer was 590nm.

The MMP assay kit (Solarbio, CHN) was used and all steps of this experiment were carried out according to the instructions. The Caco-2 cells were digested with a trypsin-EDTA solution to prepare suspensions. The Caco-2 cells were washed once with PBS. JC-1 staining solution was freshly prepared, mixed and then added to Caco-2 cells. The Caco-2 cells were placed in a cell incubator at 37 °C for 20 min. JC-1 staining buffer (5X) was diluted to 1X with deionized water and precooled on ice. At the end of incubation, the supernatant was discarded, and the Caco-2 cells were washed twice with 1X JC-1 staining buffer. Two milliliters of culture medium was added to the Caco-2 cells, which were observed and photographed under a laser confocal microscope (Red fluorescence = 590nm, and Green fluorescence = 530nm), and the electronic images were saved. ImageJ software was used to process the images, and the corresponding values were obtained and recorded.

### 5. Assessment of the apoptosis rate

The AnnexinV-FITC apoptosis detection kit (Dojindo, CHN) was used and all steps of this experiment were carried out according to the instructions. Caco-2 cells were digested with trypsin without EDTA. The cell suspension was transferred to a new 1.5 ml centrifuge tube, and the cells were collected by centrifugation at 1000 rpm/min for 3 min. The supernatant was discarded, and the cells were resuspended in 0.5 ml of PBS and collected by centrifugation at 1000 rpm/min for 3 min. The cells were resuspended in 400 µl of banding buffer (1X), and three tubes were prepared for a blank control, a FITC-positive control and a PI-positive control. In addition to the blank and PI controls, 5 µl of FITC dye was added to each tube and incubated in the dark for 15 min. In addition to the blank control and FITC control, 10 µl of PI dye was added to each tube and incubated in the dark for 5 min. The banding buffer, FITC dye and PI dye were all included in the assay kit. Cell apoptosis was subsequently assessed by flow cytometry.

### 6. Western blotting

The extraction procedure of the Caspase-9 and Caspase-3 proteins and the mitochondrial and cytoplasmic Cyto-C proteins from Caco-2 cells was performed as described in our previous study [11]. Cell mitochondria isolation kit (Beyotime, CHN) was used to obtain mitochondrial and cytoplasmic proteins for the detection of Cyto-C. Western blotting was also performed as previously described [11].

### 7. Statistical analysis

All the experimental results are expressed as the means ± standard errors (SEMs). All the statistical analyses were performed with SPSS 25.0 software. One-way ANOVA was used to compare the measurement data and Tukey’s multiple comparisons test was used to make pairwise comparisons of the resulting data. P < 0.05 indicated a significant difference, represented by *, and P < 0.01 was represented by **. All the statistical plots were generated with GraphPad Prism 8.0 software.

## Results

### 1. HSF2 inhibited the increase in mitochondrial membrane permeability in Caco-2 cells under inflammatory conditions

The experimental groups corresponding to the results in this part are as follows: a control group of normal cells (NC), an LPS-treated group of normal cells (NC + LPS), an LPS-treated group of HSF2 RNAi lentivirus-transfected cells (shR-HSF2 + LPS), an LPS-treated group of HSF2 RNAi lentiviral vector-transfected cells (shR-V + LPS), an LPS-treated group of LV-HSF2 lentivirus-transfected cells (OE-HSF2 + LPS) and an LPS-treated group of LV-HSF2 lentiviral vector-transfected cells (OE-V + LPS).

**Fig 1:**
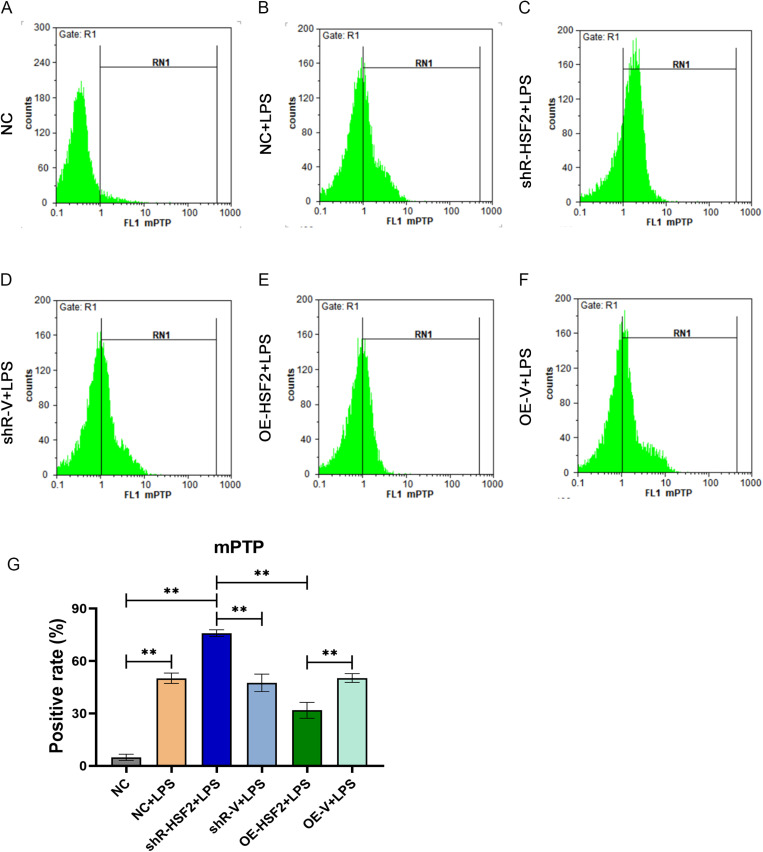
HSF2 inhibited mPTP opening in Caco-2 cells, which were detected by flow cytometry. A-F: The detection results of mPTP opening in each experimental group. G: Statistical bar chart, *P* value of less than 0.01 was identified as**. Six replicates for each group and the experiment repeated three times in the study. All the data in this study was presented as mean ± SEM.

First, flow cytometry was used to determine the degree of mPTP opening in LPS-treated Caco-2 cells expressing different levels of HSF2. Cells with an open mPTP were defined as positive cells, and the proportion of the green area within the RN1 box represented the proportion of positive cells detected. The rates of positive cells in the LPS-treated groups (the NC + LPS group, the shR-HSF2 + LPS group, the shR-V + LPS group, the OE-HSF2 + LPS group and the OE-V + LPS group) were greater than that of the non-LPS-treated group (the NC group). In the LPS-treated groups, the rate of positive cells was the highest in the shR-HSF2 + LPS group and the lowest in the OE-HSF2 + LPS group. There was no significant difference among the other three groups (the NC + LPS group, the shR-V + LPS group and the OE-V + LPS group). These results suggested that HSF2 overexpression could inhibit mPTP opening and that interference with HSF2 could increase mPTP opening in Caco-2 cells (Fig 1 and S1 Table).

**Fig 2:**
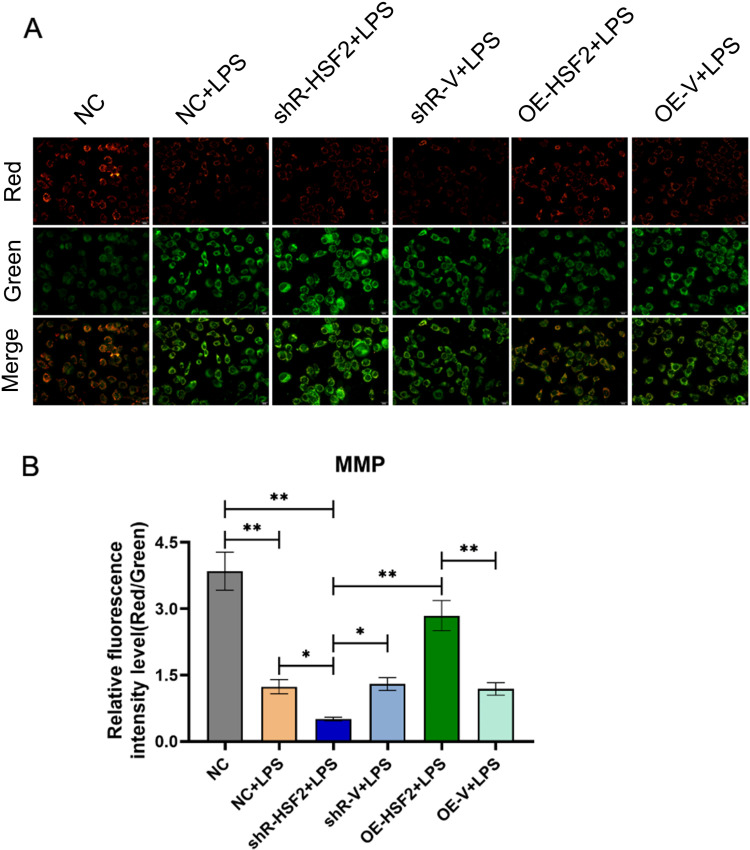
HSF2 reduced the decrease in the MMP in Caco-2 cells, which were observed by laser confocal microscopy. A: The detection results of MMP in each experimental group. Red fluorescence indicated that there was no obvious abnormality in the MMP, while green fluorescence represented a decrease in the MMP. B: Statistical bar chart of the ratio of red to green fluorescence, *P* value of less than 0.05 was identified as* and *P* value of less than 0.01 was identified as**. Six replicates for each group and the experiment repeated three times in the study. All the data in this study was presented as mean ± SEM.

Subsequently, laser confocal microscopy was used to observe the changes in the MMP in LPS-treated Caco-2 cells expressing different levels of HSF2. Red fluorescence indicated that the MMP was increased and that there was no obvious abnormality, while green fluorescence represented a decrease in the MMP. The ratios of red to green fluorescence in the LPS-treated groups were lower than that in the non-LPS-treated groups. In the LPS-treated groups, the ratio of red to green fluorescence was the highest in the OE-HSF2 + LPS group and the lowest in the shR-HSF2 + LPS group. There was no significant difference among the other three groups. These results suggested that overexpression of HSF2 could reduce the decrease in the MMP in Caco-2 cells and, conversely, that interference with HSF2 could increase the decrease in the MMP (Fig 2 and S2 Table).

The above experiments showed that HSF2 could inhibit mPTP opening, reduce the decrease in the MMP and maintain the stability of mitochondrial membrane permeability in Caco-2 cells.

### 2. HSF2 restored the stability of mitochondrial membrane permeability in Caco-2 cells after treatment with the mPTP agonist Atr

The experimental groups corresponding to the results in this part are as follows: the NC group, the NC + LPS group, the shR-HSF2 + LPS group and the OE-HSF2 + LPS group were as described above. In addition, an LPS-induced inflammation group of normal cells treated by Atr (NC + Atr + LPS), an LPS-induced inflammation group of normal cells treated by CsA (NC + CsA + LPS), an LPS-induced inflammation group of LV-HSF2 lentivirus-transfected cells treated by Atr (OE-HSF2 + Atr + LPS) and an LPS-induced inflammation group of HSF2 RNAi lentivirus-transfected cells treated by CsA (shR-HSF2 + CsA + LPS).

**Fig 3:**
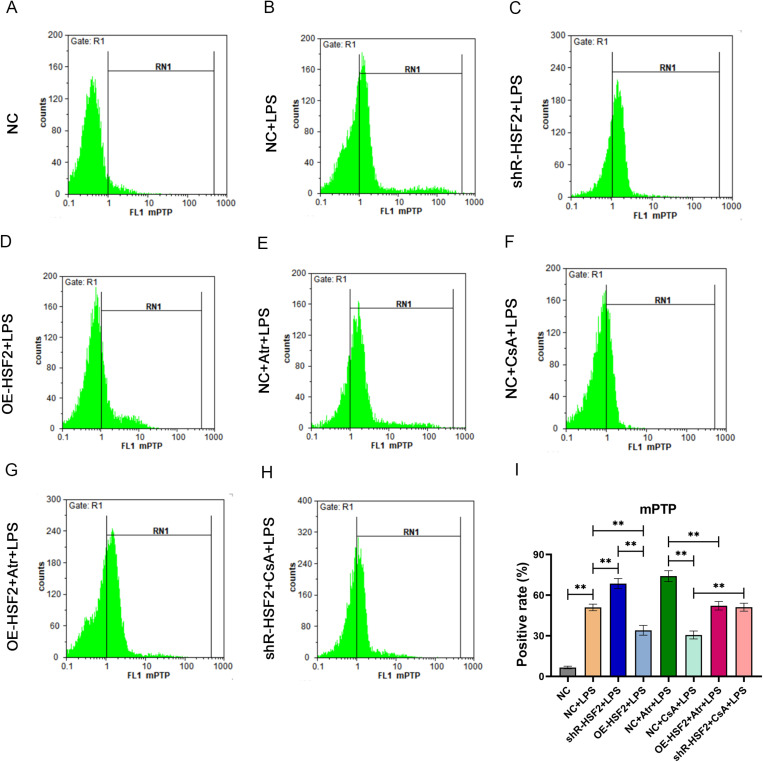
HSF2 had a similar effect as mPTP inhibitor CsA and could reverse the effect of mPTP opening induced by mPTP agonist Atr. A-H: The detection results of mPTP opening in each experimental group. I: Statistical bar chart, *P* value of less than 0.01 was identified as**. Six replicates for each group and the experiment repeated three times in the study. All the data in this study was presented as mean ± SEM.

In these experiments, the mPTP agonist Atr and inhibitor CsA were used to treat Caco-2 cells to clarify the regulatory effect of HSF2 on the mPTP. The degree of mPTP opening in the Caco-2 cells in each experimental group was assessed by flow cytometry. The results revealed that there was no significant difference in the percentage of mPTP-open cells between the NC + CsA + LPS group and the OE-HSF2 + LPS group, and the percentage in both groups was lower than that in the NC + LPS group. There was also no significant difference in the percentage of positive cells between the NC + Atr + LPS group and the shR-HSF2 + LPS group, and the percentage of positive cells in both groups was greater than that in the NC + LPS group. Moreover, the percentage of positive cells in the OE-HSF2 + Atr + LPS group was lower than that in the NC + Atr + LPS group, and the percentage of positive cells in the shR-HSF2 + CsA + LPS group was greater than that in the NC + CsA + LPS group (Fig 3 and S3 Table).

**Fig 4:**
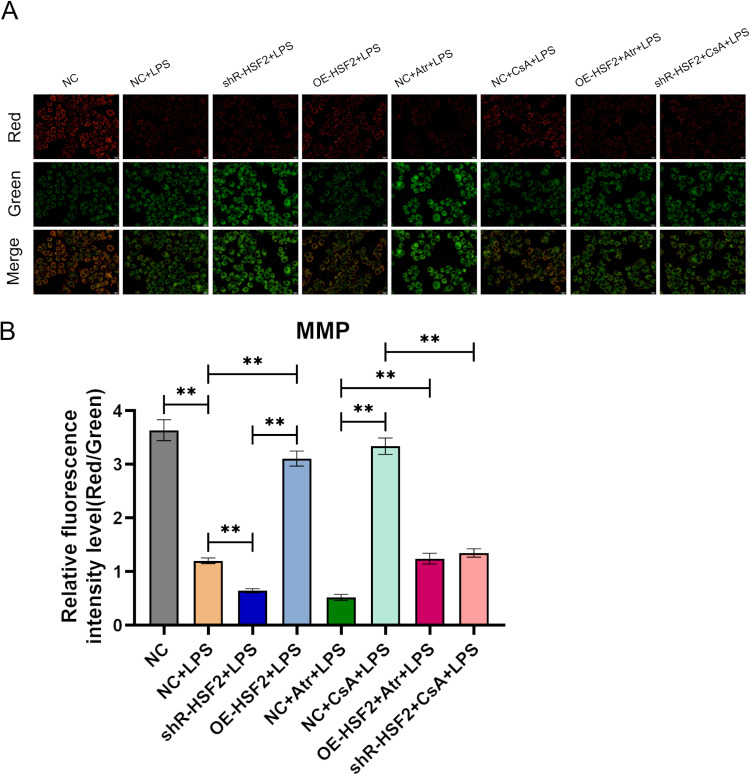
HSF2 reversed the decrease in the MMP induced by Atr. A: The detection results of MMP in each experimental group. B: Statistical bar chart of the ratio of red to green fluorescence, *P* value of less than 0.01 was identified as**. Six replicates for each group and the experiment repeated three times in the study. All the data in this study was presented as mean ± SEM.

Next, changes in the MMP of Caco-2 cells in each experimental group were observed with laser confocal microscopy. The results revealed that there was no significant difference in the ratios of red to green fluorescence between the NC + CsA + LPS group and the OE-HSF2 + LPS group, and the ratios in both groups were greater than that in the NC + LPS group. The overexpression of HSF2 and CsA both could reduce the decrease in the MMP in Caco-2 cells under inflammatory condition. The ratios were also not significantly different between the NC + Atr + LPS group and the shR-HSF2 + LPS group, and the ratios in both groups were lower than that in the NC + LPS group. The interference with HSF2 and Atr both could increase the decrease in the MMP in Caco-2 cells under inflammatory condition. The ratio in the OE-HSF2 + Atr + LPS group was greater than that in the NC + Atr + LPS group, whereas the ratio in the shR-HSF2 + CsA + LPS group was lower than that in the NC + CsA + LPS group (Fig 4 and S4 Table).

The above experiments indicated that the overexpression of HSF2 and CsA had similar effects on the inhibition of mPTP opening and the decrease in MMP. In contrast, the effect of HSF2 interference was similar to that of Atr. In addition, the overexpression of HSF2 reversed the effects of the opening of the mPTP and the decrease in the MMP induced by Atr and restored the stability of mitochondrial membrane permeability.

### 3. The protective effect of HSF2 on inhibiting the mitochondrial pathway of apoptosis in Caco-2 cells after treatment with the mPTP agonist Atr

The experimental groups corresponding to the results in this part are as follows: the NC group, the NC + LPS group, the shR-HSF2 + LPS group, the OE-HSF2 + LPS group, the NC + Atr + LPS group, the NC + CsA + LPS group, the OE-HSF2 + Atr + LPS group and the shR-HSF2 + CsA + LPS group.

**Fig 5:**
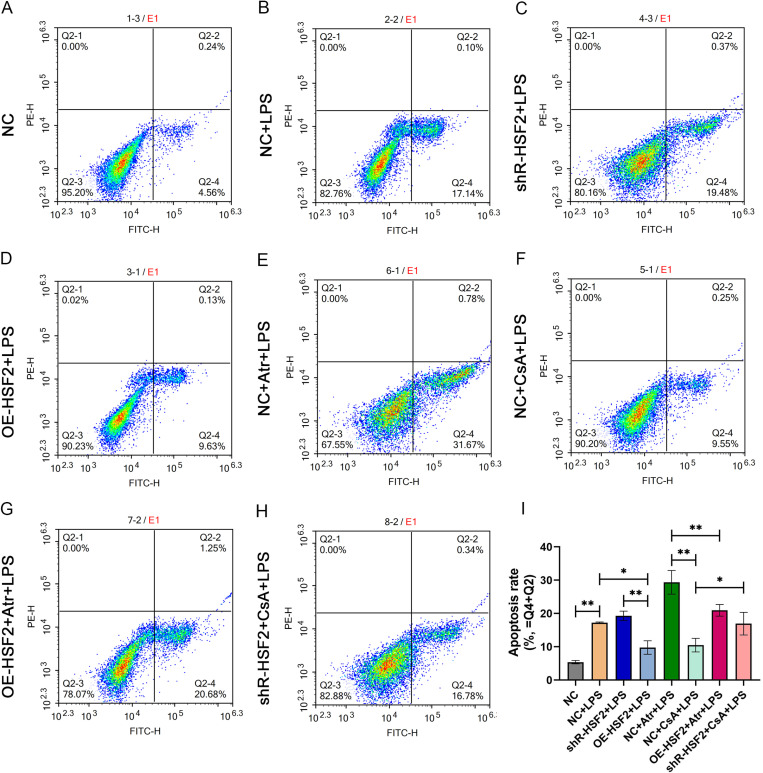
HSF2 decreased the apoptosis rate of Caco-2 cells treated by Atr. A-H: The detection results of cell apoptosis rate in each experimental group. I: Statistical bar chart of apoptosis rate, *P* value of less than 0.05 was identified as* and *P* value of less than 0.01 was identified as**. Six replicates for each group and the experiment repeated three times in the study. All the data in this study was presented as mean ± SEM.

In the final series of experiments, Atr and CsA were used to clarify the regulatory effects of HSF2 on the mitochondrial pathway of apoptosis. The apoptosis rate in the Caco-2 cells of each experimental group was assessed by flow cytometry. The results revealed that there was no significant difference in the apoptosis rate between the NC + CsA + LPS group and the OE-HSF2 + LPS group, and the rates of both groups were lower than that in the NC + LPS group. The apoptosis rate in both the NC + Atr + LPS group and the shR-HSF2 + LPS group were greater than that in the NC + LPS group. Furthermore, the apoptosis rate in the OE-HSF2 + Atr + LPS group was lower than that in the NC + Atr + LPS group, and the apoptosis rate in the shR-HSF2 + CsA + LPS group was greater than that in the NC + CsA + LPS group (Fig 5 and S5 Table).

Through the above experiment, we found that both HSF2 and CsA could reduce the apoptosis rate of Caco-2 cells under inflammatory conditions. Meanwhile, through the second part of the experiments, we found that HSF2 and CsA had a similar effect in inhibiting mPTP opening. To further clarify the protective mechanism of HSF2, the western blotting experiments were continued to explore the effect of HSF2 on the mitochondrial pathway apoptosis level of Caco-2 cells after acting on mPTP. The OE-HSF2 + LPS group and the NC + CsA + LPS group were observed to clarify the effect of mPTP inhibition on the mitochondrial pathway apoptosis level of Caco-2 cells. The NC + Atr + LPS group and the OE-HSF2 + Atr + LPS group were compared, aiming to confirm the effect of HSF2 in inhibiting mPTP opening and reducing mitochondrial pathway apoptosis levels in Caco-2 cells through rescue experiments.

**Fig 6:**
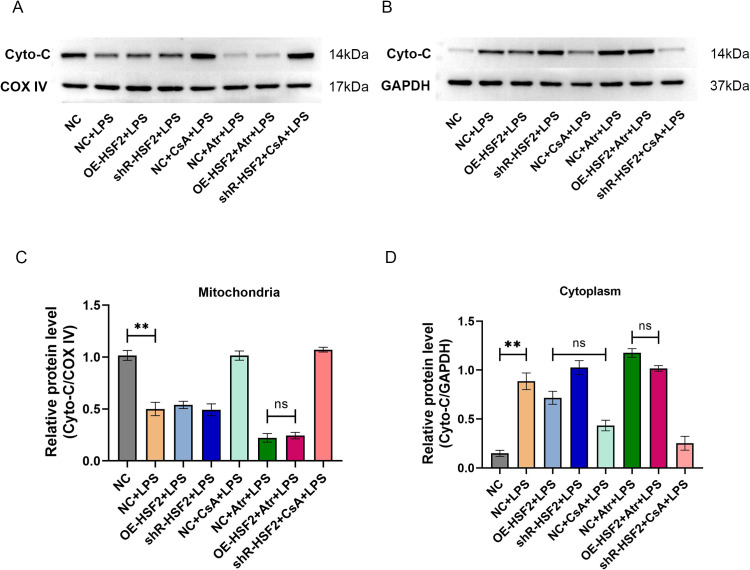
Expression levels of Cyto-C protein in mitochondria and cytoplasm of each experimental group. A and B: The visualized protein bands of Cyto-C, COX IV and GAPDH were obtained by western blotting. C and D: Statistical bar chart of the relative protein levels, *P* value of less than 0.01 was identified as** and *P* value of more than 0.05 was identified as ns. Six replicates for each group and the experiment repeated three times in the study. All the data in this study was presented as mean ± SEM.

The expression levels of the mitochondrial apoptosis pathway-related proteins Cyto-C, Caspase-9 and Caspase-3 in Caco-2 cells from each experimental group were subsequently determined by western blotting. First, mitochondrial and cytoplasmic proteins were isolated for Cyto-C detection. The results revealed that the expression levels of the mitochondrial Cyto-C protein were the highest in the NC group and the CsA treatment groups (the NC + CsA + LPS group and the shR-HSF2 + CsA + LPS group), and there was no statistically significant difference between the groups. The expression level of the mitochondrial Cyto-C protein was the lowest in the Atr treatment groups (the NC + Atr + LPS group and the OE-HSF2 + Atr + LPS group), and there was no significant difference between the groups, although the level was slightly greater in the OE-HSF2 + Atr + LPS group than in the NC + Atr + LPS group. The overall expression of the cytoplasmic Cyto-C protein showed the opposite trend. Notably, the expression levels of cytoplasmic Cyto-C protein were not significantly different between the OE-HSF2 + LPS group and the NC + CsA + LPS group. Moreover, although there was no significant difference between the levels in the OE-HSF2 + Atr + LPS group and the NC + Atr + LPS group, the level was slightly lower in the OE-HSF2 + Atr + LPS group than in the NC + Atr + LPS group (Fig 6).

**Fig 7:**
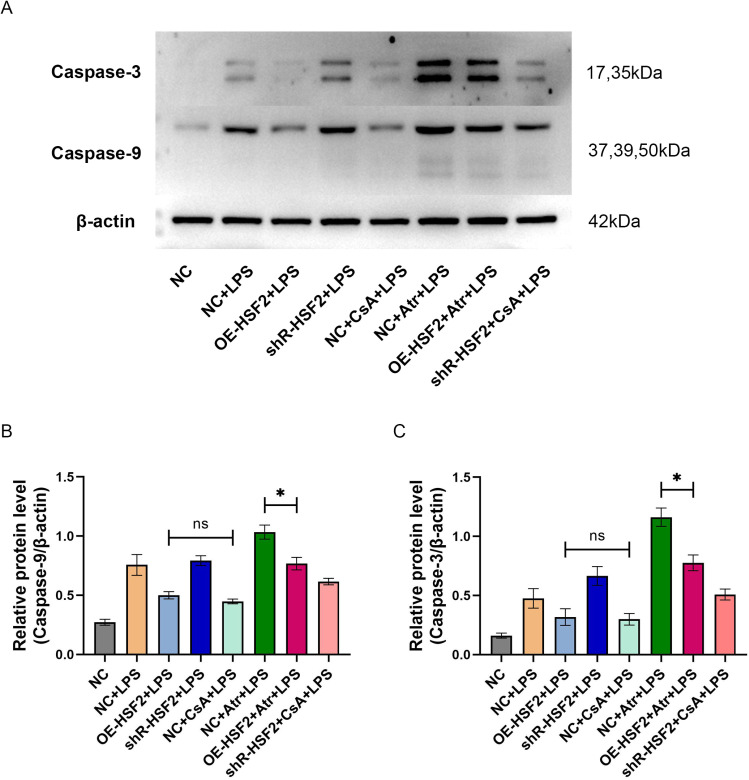
Expression levels of Caspase-9 and Caspase-3 proteins in each experimental group. A: The visualized protein bands of Caspase-9, Caspase-3 and β-actin were obtained by western blotting. B and C: Statistical bar chart of the relative protein levels, *P* value of less than 0.05 was identified as* and *P* value of more than 0.05 was identified as ns. Six replicates for each group and the experiment repeated three times in the study. All the data in this study was presented as mean ± SEM.

The protein expression levels of Caspase-9 and Caspase-3 were also detected by western blotting in these experiments. The results revealed that there was no statistically significant difference in either the Caspase-9 or the Caspase-3 protein levels between the OE-HSF2 + LPS group and the NC + CsA + LPS group. However, there was a statistically significant difference in both the Caspase-9 and Caspase-3 protein levels between the OE-HSF2 + Atr + LPS group and the NC + Atr + LPS group. The protein expression levels of both Caspase-9 and Caspase-3 were lower in the OE-HSF2 + Atr + LPS group than in the NC + Atr + LPS group (Fig 7).

These findings confirmed that HSF2 could inhibit mitochondrial pathway of apoptosis in Caco-2 cells by affecting the mPTP.

## Discussion

UC is a chronic inflammatory disease of the intestine with an unknown etiology. Our research has focused on the role and mechanism of HSF2 in the occurrence and development of UC for many years. HSF2 was found to be highly expressed [8], promoting mitophagy, and reducing mitochondria damage in UC [12]. The phenomenon of apoptosis in IECs was observed under electron microscope (S1 Fig). In addition, HSF2 inhibits IECs mitochondrial pathway apoptosis in UC, but the mechanism is not clear [11]. This study focused on the relationship between mitochondrial membrane permeability and the mitochondrial pathway of apoptosis in IECs and explored the protective mechanism of HSF2 in UC.

First, we examined the changes in mitochondrial membrane permeability, including the degree of mPTP opening and the change in the MMP, due to the expression of different levels of HSF2 in a Caco-2 cell model of inflammation induced by LPS. The flow cytometry results revealed that the level of HSF2 in Caco-2 cells was inversely proportional to the degree of mPTP opening. The overexpression of HSF2 inhibited mPTP opening, and interference with HSF2 promoted mPTP opening. The laser confocal microscopy results revealed that the level of HSF2 was proportional to the ratio of red to green fluorescence. The MMP in the inflammatory HSF2 interference group (the shR-HSF2 + LPS group) was lower than that in the inflammatory control group (the NC + LPS group), whereas the MMP in the inflammatory HSF2 overexpression group (the OE-HSF2 + LPS group) was greater than that in the inflammatory control group (the NC + LPS group). The above results indicated that the level of HSF2 in Caco-2 cells was inversely proportional to mitochondrial membrane permeability. Interference with HSF2 increased the degree of mPTP opening, leading to a decrease in the MMP, whereas overexpression of HSF2 led to the opposite effect. Liang J et al. reported that a decrease in HSF1 in mouse kidney cells promoted mPTP opening [21]. Moreover, another study revealed that the degree of mPTP opening in the heart tissue of HSF1 knockout mice was increased [22]. HSF2 is highly homologous to HSF1 [23]. In this study, we also revealed that interfering with the level of HSF2 in Caco-2 cells promoted mPTP opening, which indicated a role similar to that of HSF1.

Second, the mPTP agonist Atr and inhibitor CsA were used to treat Caco-2 cells, and the two aforementioned experiments were conducted again to further verify the effect of HSF2 on the mPTP. The flow cytometry results revealed that interference with HSF2 promoted mPTP opening and that the effect was similar to that of Atr. The overexpression of HSF2 inhibited mPTP opening, and the effect was similar to that of CsA. Moreover, the degree of mPTP opening in the shR-HSF2 + CsA + LPS group was greater than that in the NC + CsA + LPS group, and that in the OE-HSF2 + Atr + LPS group was lower than that in the NC + Atr + LPS group. These results confirmed that the level of HSF2 in Caco-2 cells was inversely proportional to the degree of mPTP opening. In addition, HSF2 had a restorative effect on the opening of the mPTP induced by Atr. The statistical analysis of the experimental results from the laser confocal microscopy also led to similar conclusions. The HSF2 levels in Caco-2 cells were directly proportional to the MMP. Moreover, HSF2 reversed the decrease in the MMP induced by Atr.

Third, experiments were performed to explore the regulatory effect of HSF2-mediated inhibition of mPTP opening on the mitochondrial pathway of apoptosis in IECs. In the first experiment, flow cytometry was used to assess the apoptosis rate of the Caco-2 cells in each experimental group. The results revealed that the apoptosis rate of cells in the OE-HSF2 + Atr + LPS group was significantly lower than that in the NC + Atr + LPS group. In the second experiment, western blotting was used to determine the expression levels of the mitochondrial apoptotic pathway proteins Cyto-C, Caspase-9 and Caspase-3 in the Caco-2 cells of each experimental group. According to the distribution characteristics of Cyto-C in cells, the expression levels of Cyto-C in the mitochondria and cytoplasm were separately assessed. Although there was no statistically significant difference in the expression level of mitochondrial Cyto-C between the NC + Atr + LPS group and the OE-HSF2 + Atr + LPS group, the level of the latter was slightly greater than that of the former. The opposite result was observed for the expression level of Cyto-C in the cytoplasm. In addition, there was no significant difference in the expression levels of Caspase-9 and Caspase-3 between the OE-HSF2 + LPS group and the NC + CsA + LPS group, and the level in the OE-HSF2 + Atr + LPS group was significantly lower than that in the NC + Atr + LPS group. These results indicated that the inhibitory effect of HSF2 on mPTP opening could reduce Caco-2 cell apoptosis through the mitochondrial pathway.

Our study reveals the following breakthrough findings. First, we confirmed for the first time that HSF2 inhibits mPTP opening, maintains the MMP, counteracts the increase in mitochondrial membrane permeability, and plays a role similar to that of the mPTP inhibitor CsA. Notably, CsA is an effective drug commonly used in the clinical treatment of UC [24], which indirectly indicates that inhibiting mPTP opening may be beneficial for the remission of UC. Therefore, HSF2, which has a similar inhibitory effect on the mPTP as CsA does, is also expected to become a potential therapeutic molecule for UC, providing new ideas for the clinical treatment of UC. Second, for the first time, we revealed that the inhibitory effect of HSF2 on mPTP opening can effectively reduce the mitochondrial pathway of apoptosis in IECs.

However, there are several shortcomings in this study. The structure and state of the mPTP are also important for its opening. The mPTP is composed of three main components: adenine nucleotide translocase (ANT), which is located in the inner membrane of mitochondria; voltage-dependent anion selective channel (VDAC), which is located in the outer membrane of mitochondria; and cyclophilin D (Cyp D), which is located in the matrix [25]. Previous studies reported that HSPs regulate mPTP opening by interacting with mPTP components. HSP70 in mitochondria, namely, mt-HSP70, can directly interact with VDAC to regulate mPTP opening and mitochondrial pathway apoptosis [26]. In addition, the HSP90 family members can affect mPTP opening by interacting with Cyp D proteins [27], such as HSP75. Therefore, in future work, it will be necessary to explore the role and specific mechanism of HSF2 involving these three components of the mPTP, with a focus on changes in expression, conformational changes and protein modifications of the three components due to HSF2 activity. At the same time, it should be clarified whether the inhibitory effect of HSF2 on the mPTP is achieved by HSF2 first acting on HSPs.

## Conclusions

In conclusion, this study revealed for the first time that HSF2 reduces mitochondrial pathway apoptosis in IECs by inhibiting mPTP opening, maintaining the MMP, and opposing the increase in mitochondrial membrane permeability.

## Supporting information

S1 Table Raw data, statistical analysis and description of Fig 1. (XLSX)

S2 Table Raw data, statistical analysis and description of Fig 2. (XLSX)

S3 Table Raw data, statistical analysis and description of Fig 3. (XLSX)

S4 Table Raw data, statistical analysis and description of Fig 4. (XLSX)

S5 Table Raw data, statistical analysis and description of Fig 5. (XLSX)

S1 Fig Electron microscope images of apoptosis. (PDF)

S2 Fig Raw images of western blot. (PDF)
