# Supplementary figures and images for: Heat shock transcription factor 2 reduces mitochondrial pathway apoptosis in intestinal epithelial cells by inhibiting the increase in mitochondrial membrane permeability in ulcerative colitis
Source: PLoS One. 2025 May 29;20(5):e0325275. doi: 10.1371/journal.pone.0325275 (PMC12121780; doi:10.1371/journal.pone.0325275)

A

NC

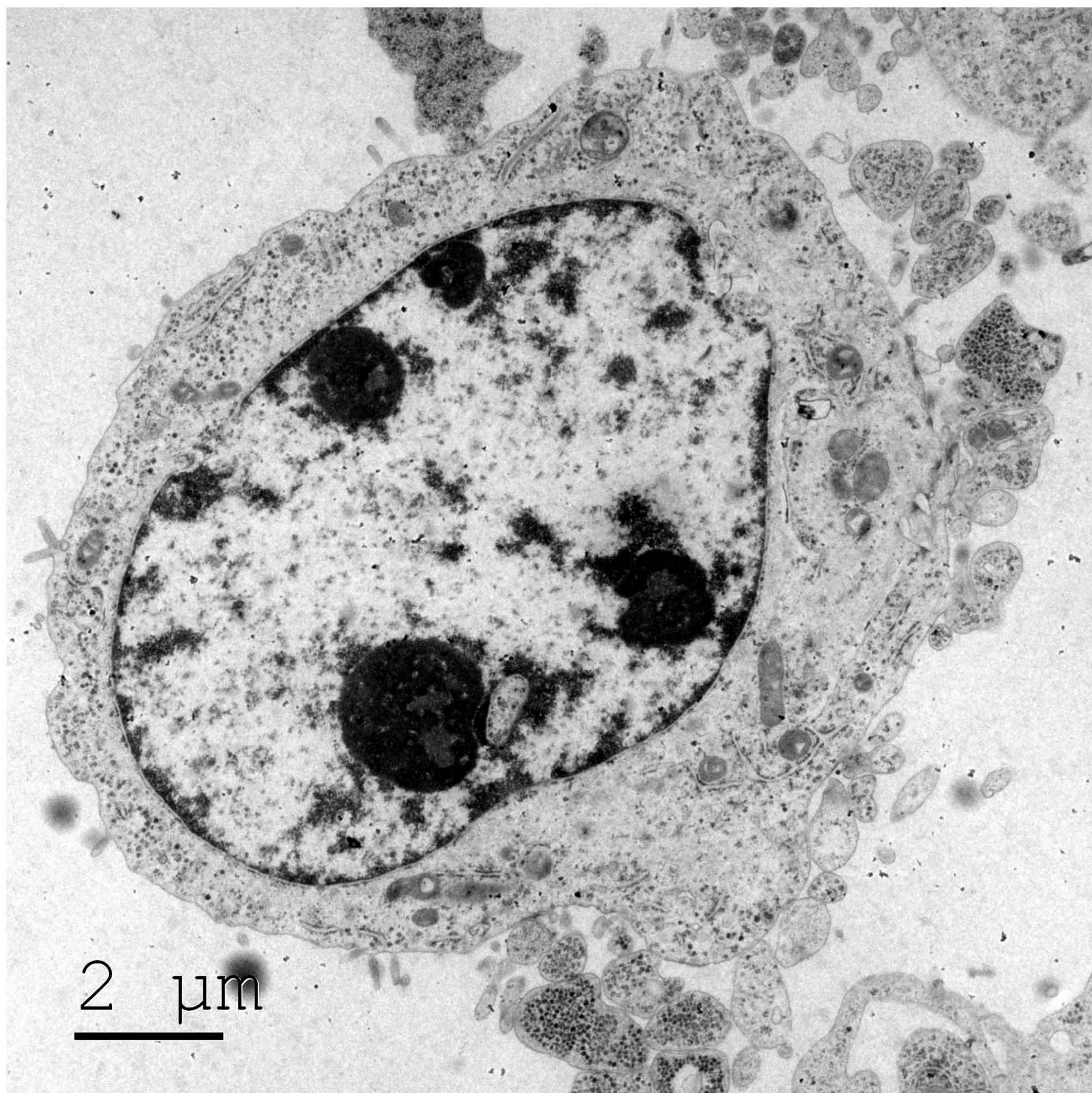

B

NC+LPS

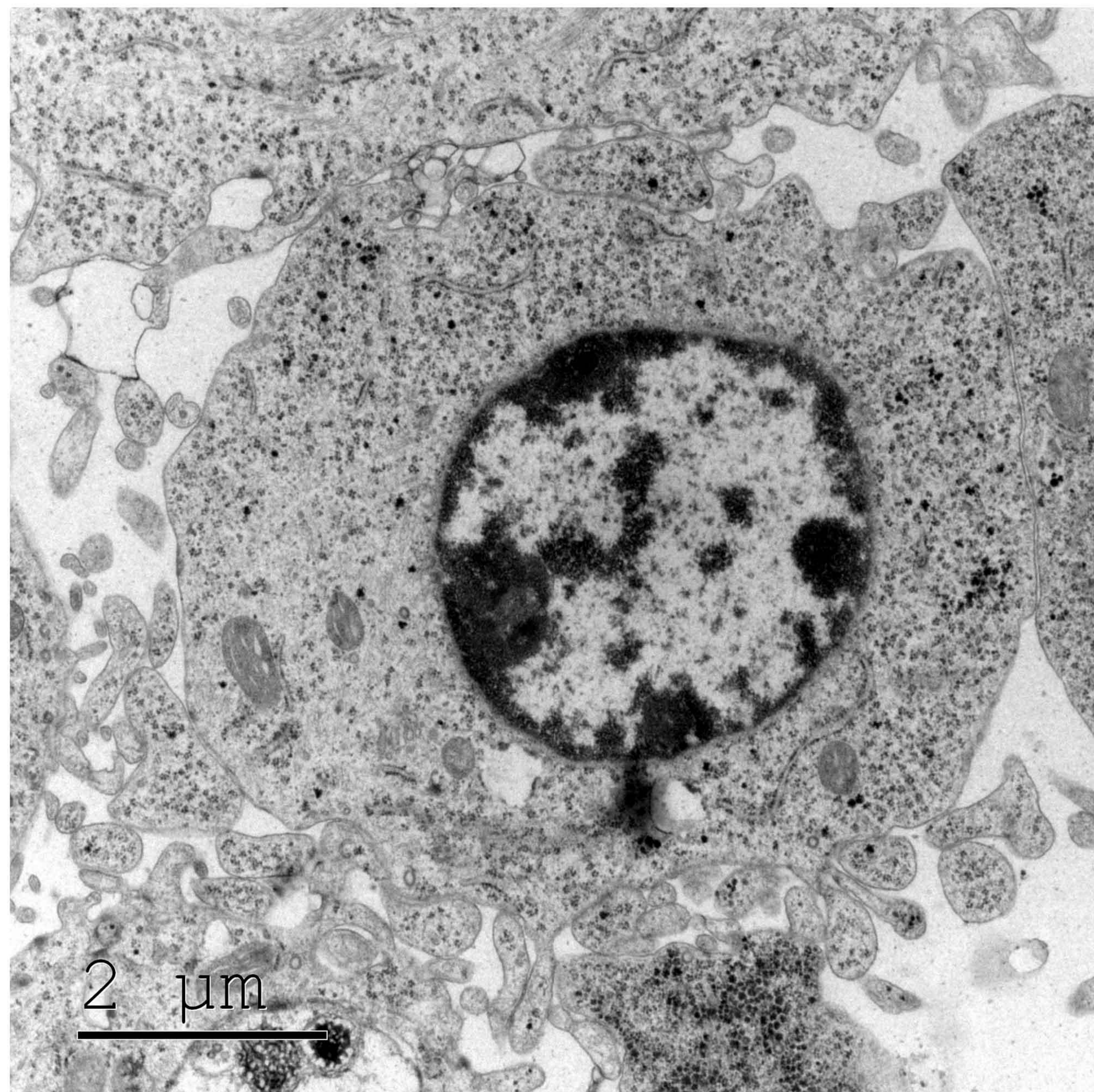

C

shR-HSF2+LPS

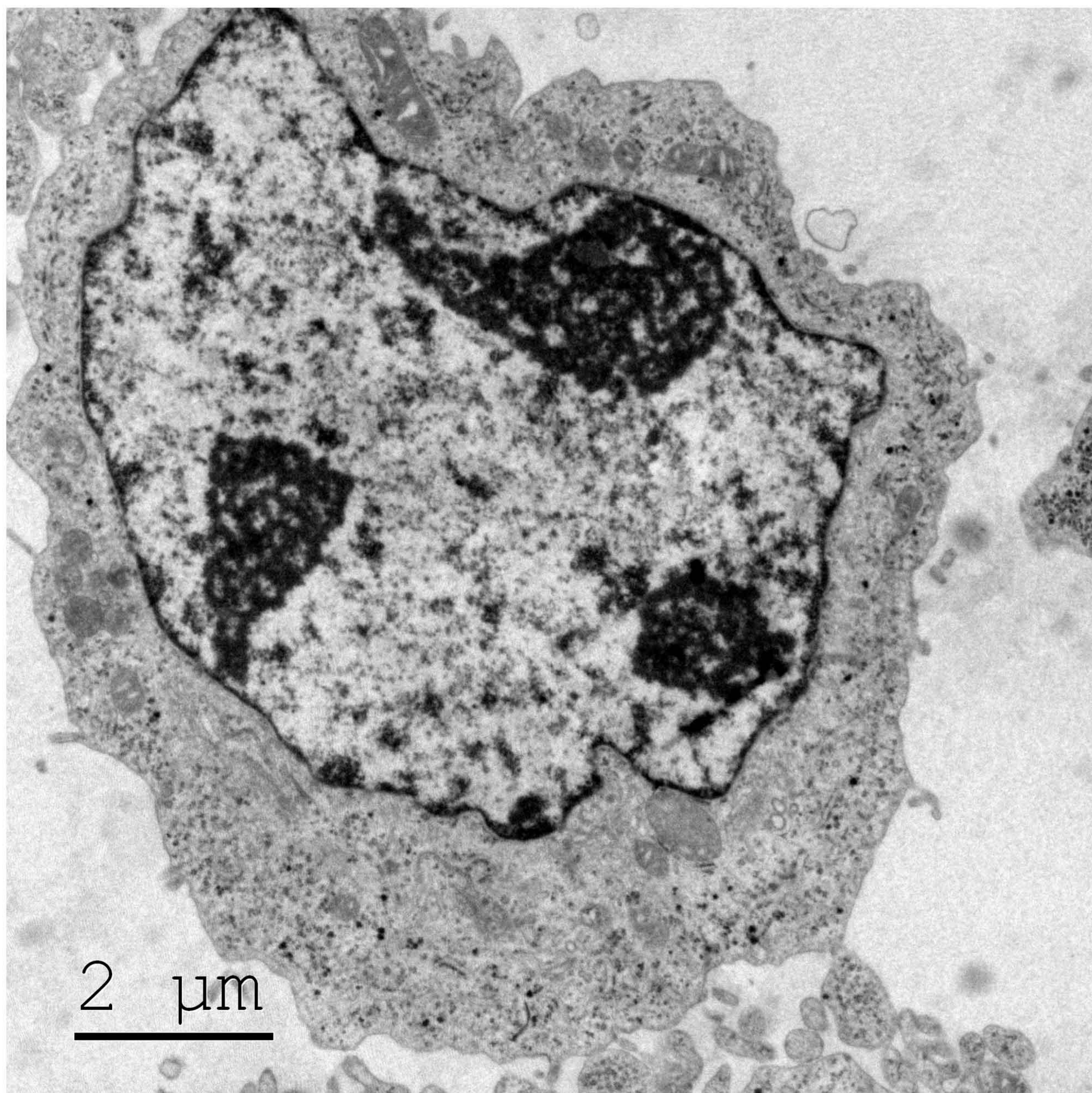

D

shR-V+LPS

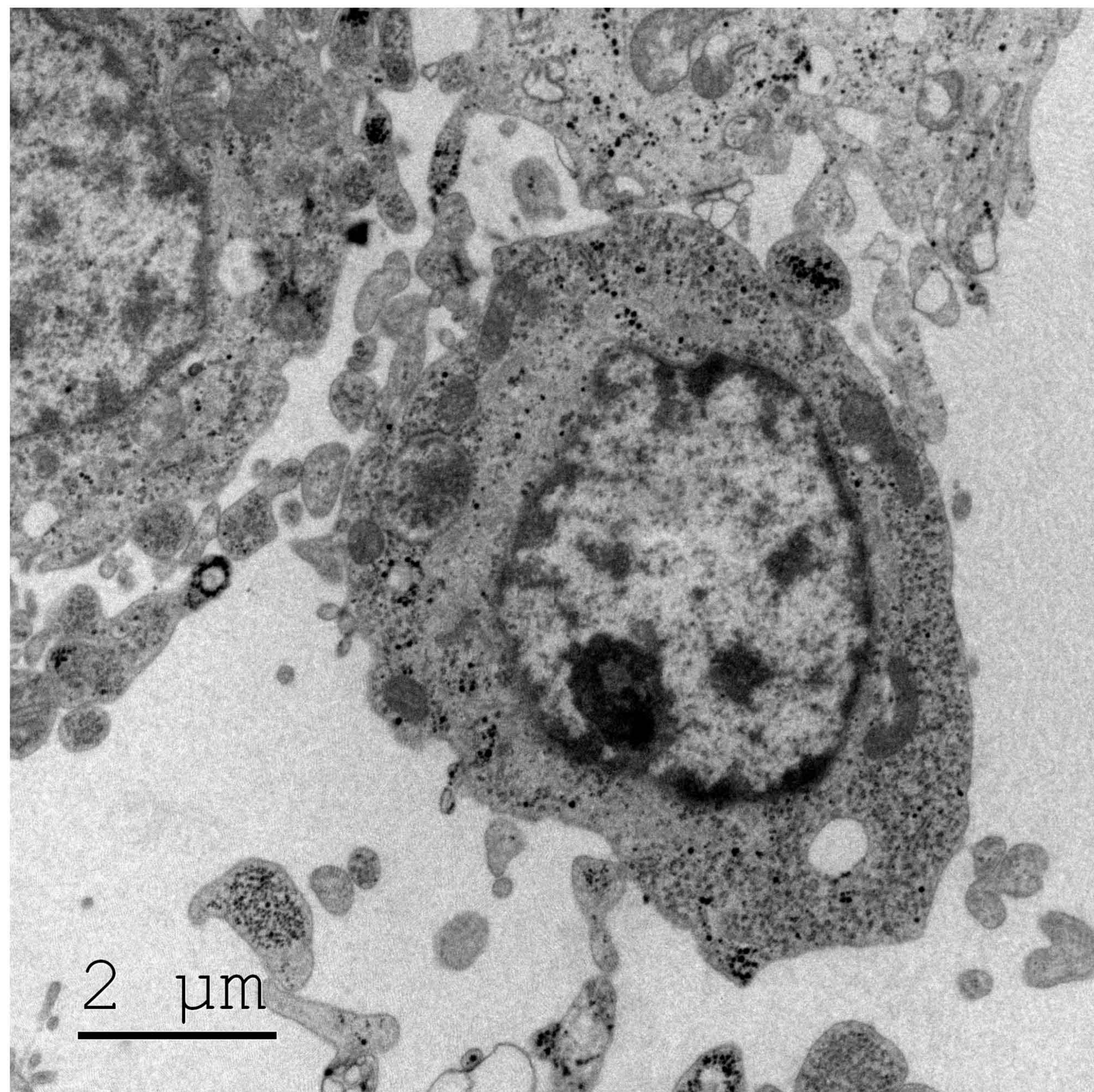

E

OE-HSF2+LPS

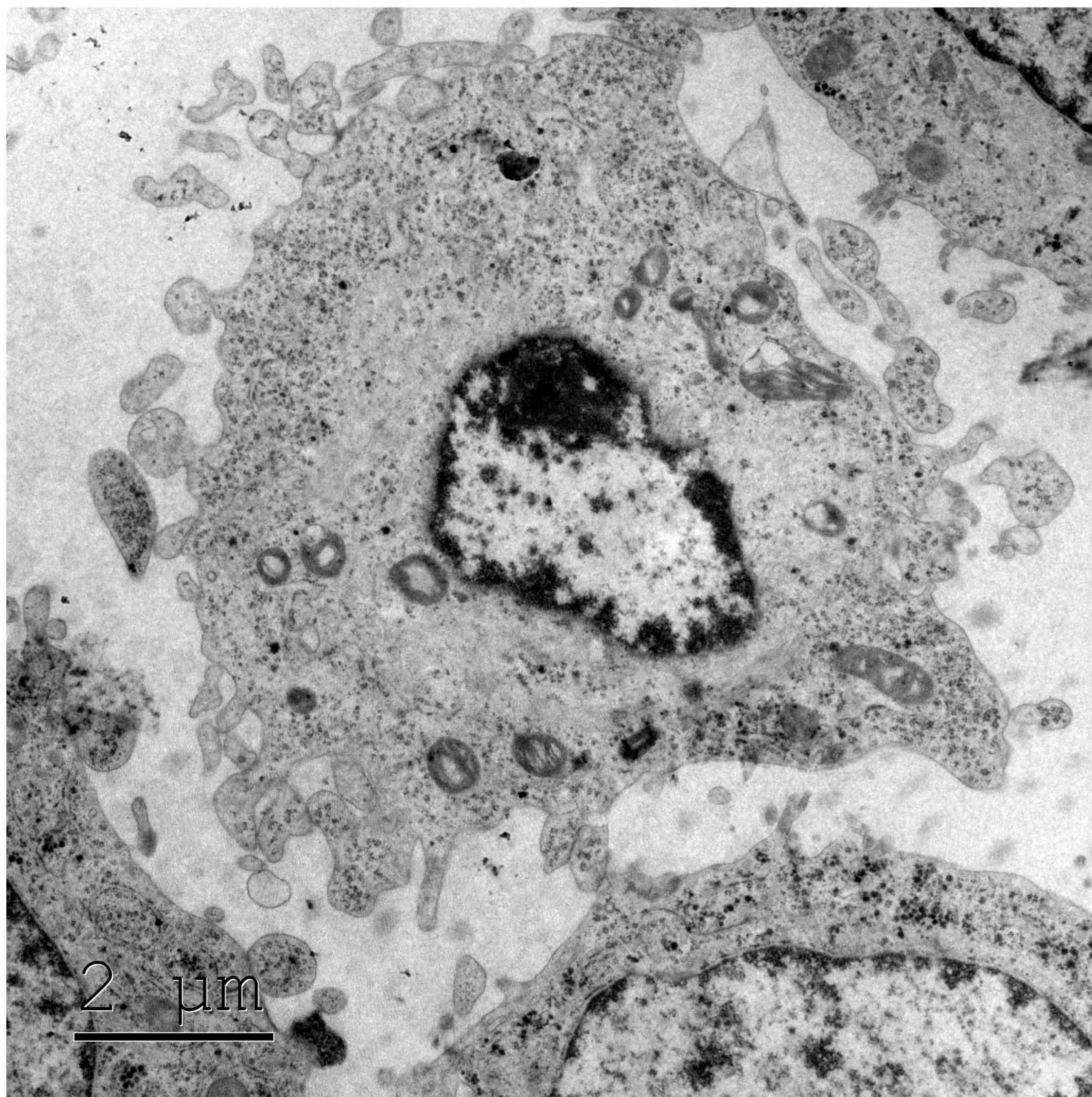

F

OE-V+LPS

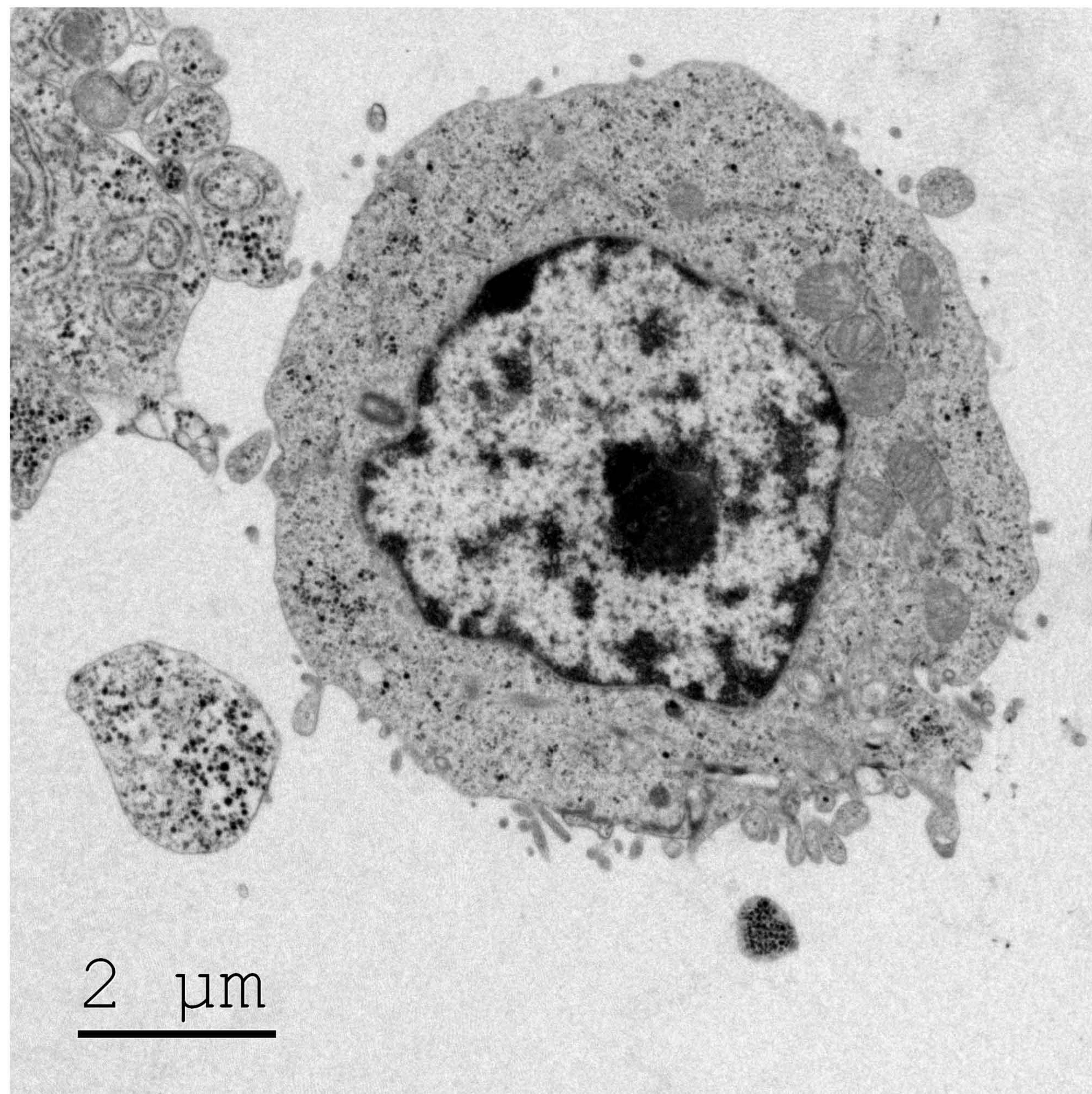

Supplement: S1 Fig — (PDF) [file pone.0325275.s006.pdf]

Fig 6

A

(1) Cyto-C

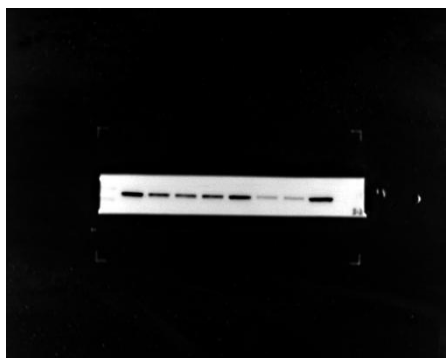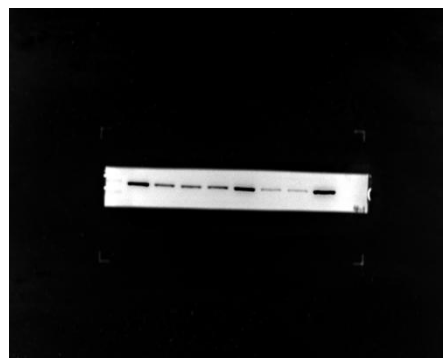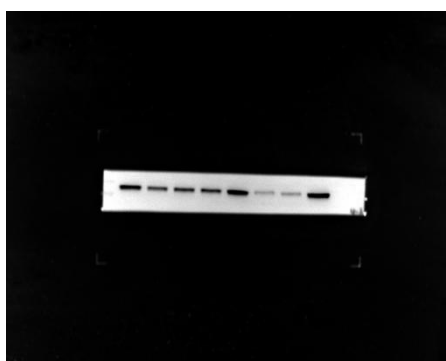

(2) Cox IV

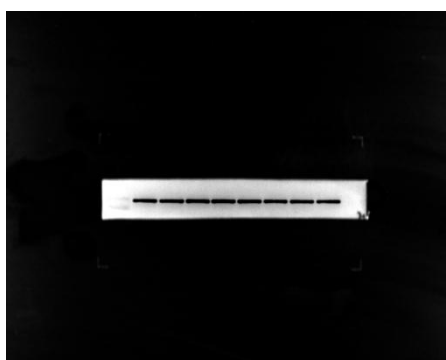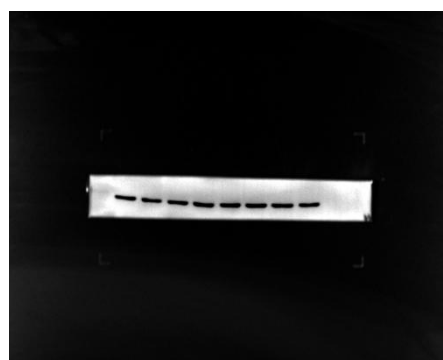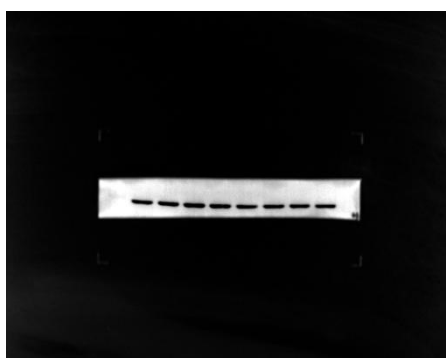

B

(1) Cyto-C

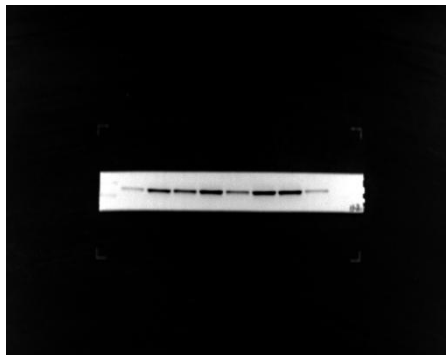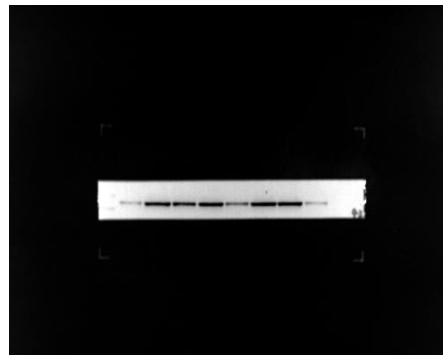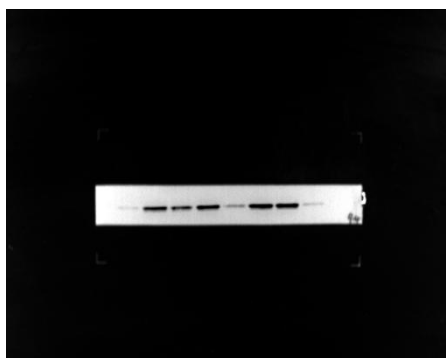

(2) GAPDH

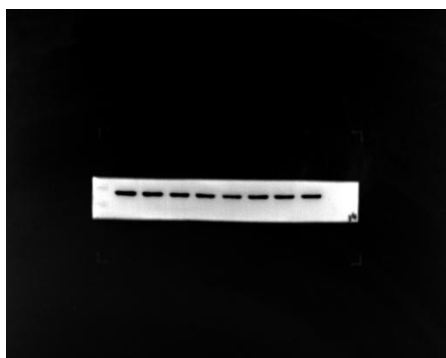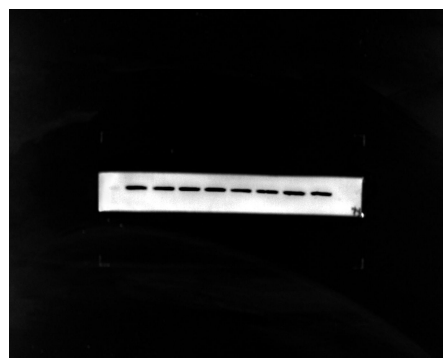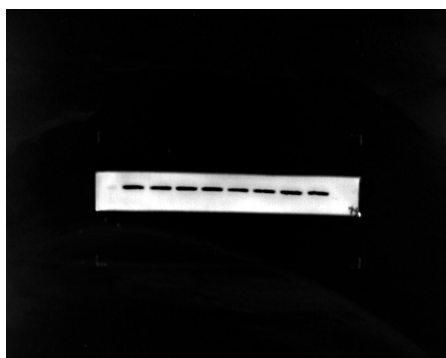

Fig 7

A

(1) Caspase-3

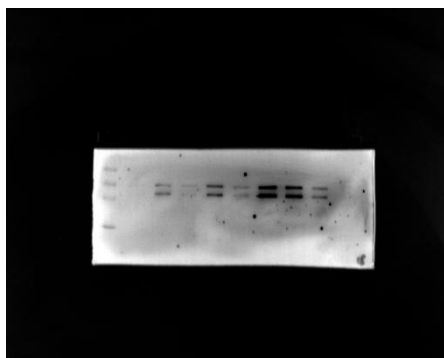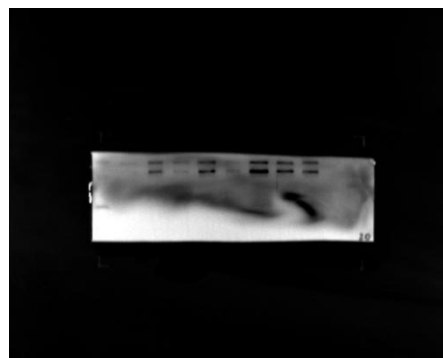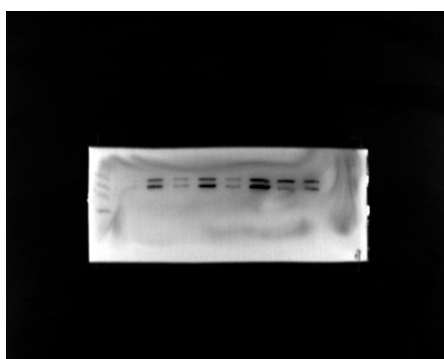

(2) Caspase-9

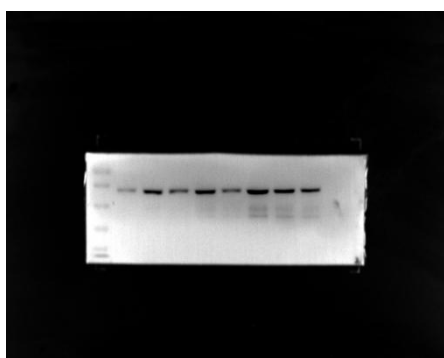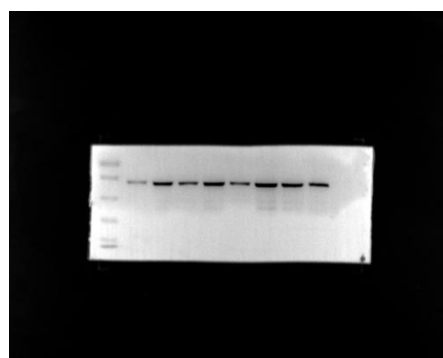

(3)  $\beta$ -actin

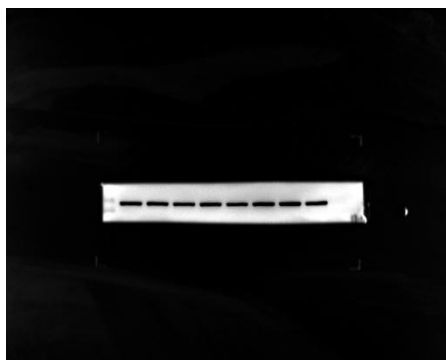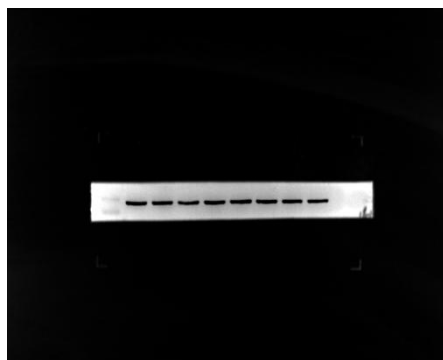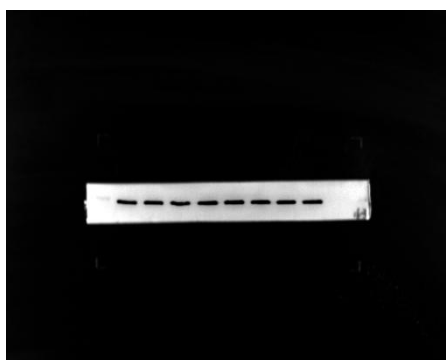

Supplement: S2 Fig — (PDF) [file pone.0325275.s007.pdf]
